# Chronic Kidney Disease and Nonalcoholic Fatty Liver Disease—Is There a Link?

**DOI:** 10.1155/2014/847539

**Published:** 2014-03-06

**Authors:** L. Orlić, I. Mikolasevic, Z. Bagic, S. Racki, D. Stimac, S. Milic

**Affiliations:** ^1^Division of Internal Medicine, Department of Nephrology and Dialysis, University Hospital Center Rijeka, 51000 Rijeka, Croatia; ^2^Division of Internal Medicine, Department of Gastroenterology, University Hospital Center Rijeka, 51000 Rijeka, Croatia

## Abstract

Research in recent years has led to the recognition of the importance of nonalcoholic fatty liver disease (NAFLD) and its relationship to the metabolic syndrome (MS). This has led to a growing interest in the potential prognostic value of NAFLD for adverse cardiovascular disease (CVD) outcome. On the other hand, searching for new risk factors for chronic kidney disease (CKD) development and progression is very important. Growing evidence suggests that the MS is an important factor in the pathogenesis of CKD. The best confirmation of this pathogenic link is hypertensive and diabetic nephropathy as the main causes of CKD. Furthermore, the possible link between NAFLD and CKD has also attracted research interest and recent data suggest an association between these two conditions. These findings have fuelled concerns that NAFLD may be a new and added risk factor for the development and progression of CKD. NAFLD and CKD share some important cardiometabolic risk factors and possible common pathophysiological mechanisms, and both are linked to an increased risk of incident CVD events. Therefore, common factors underlying the pathogenesis of NAFLD and CKD may be insulin resistance, oxidative stress, activation of rennin-angiotensin system, and inappropriate secretion of inflammatory cytokines by steatotic and inflamed liver.

## 1. Introduction

Chronic kidney disease (CKD) is a worldwide health problem and according to data from the United States population-based Third National Health and Nutrition Examination Survey (NHANES III) the prevalence of CKD in the United States is approximately 13% [1]. In Europe, the prevalence of CKD is very similar to the United States. Furthermore, according to recent data, over 1.1 million patients are estimated to have end-stage-renal disease (ESRD) worldwide. Today, it is well known that cardiovascular diseases (CVD) are the leading cause of morbidity and mortality in CKD patients and they are responsible for almost 40% of hospitalizations and 50% of deaths in these patients. Less than a half of CKD patients develop ESRD and most of them die from arteriosclerotic CVD before they develop ESRD and need for renal replacement therapy [2–7].

Nonalcoholic fatty liver disease (NAFLD) is one of the most common forms of liver disease in Western countries. Depending on the diagnostic criteria used the prevalence of NAFLD ranges from 10% to 24% in the general population. The prevalence of NAFLD increases with age, from less than 20% in people under the age of 20 to more than 40% in people over 60s. It occurs in people who do not consume large amounts of alcohol. The clinical spectrum of NAFLD ranges from simple steatosis to nonalcoholic steatohepatitis (NASH), cirrhosis, and hepatocellular carcinoma [8–11]. Today, it is believed that NAFLD is a hepatic manifestation of metabolic syndrome (MS) and thus is closely related to cardiovascular morbidity and mortality. According to the literature, in the great majority of cases, NAFLD arises in association with either one or more features of the metabolic syndrome, namely, insulin resistance, diabetes mellitus, central obesity, dyslipidemia, and hypertension. Furthermore, recent data supports the hypothesis that NAFLD itself might contribute to a higher risk of CVD independent of other prognostic risk factors. Also, novel findings support the possibility that NAFLD and atherosclerosis share common molecular mediators and that NAFLD is not merely a marker but also an early mediator of atherosclerosis [10, 12, 13].

As mentioned above, researches in recent years have led to the recognition of the importance of NAFLD and its relationship to the MS. This has led to a growing interest in the potential prognostic value of NAFLD for adverse CVD outcome [14]. On the other hand, searching for new risk factors for CKD development and progression is very important. Furthermore, the possible link between NAFLD and CKD has also attracted research interest and recent data suggest an association between these two conditions. These findings have fuelled concerns that NAFLD may be a new and added risk factor for the development and progression of CKD. NAFLD and CKD share some important cardiometabolic risk factors and possible common pathophysiological mechanisms, and both are linked to an increased risk of incident CVD events [15–18]. Moreover, the pathophysiological link between the liver and kidney is well known in some patients with decompensated liver cirrhosis manifesting as hepatorenal syndrome. This review will focus on the recent clinical evidence suggesting the link between NAFLD and CKD and how this association may lead to deterioration in renal function.

## 2. A Prevalence of Risk Factors of Chronic Kidney Disease in Patients with NAFLD

As mentioned above, patients with NAFLD often meet the diagnostic criteria for the MS. Approximately 90% of patients with NAFLD have more than one component of MS; 35–75% meet the diagnostic criteria. Furthermore, NAFLD has recently been described as an independent factor for development of diabetes mellitus type 2, independent from MS. Consequently, patients with NAFLD have multiple risk factors for CVD. The presence of insulin resistance (IR) is recognized as the pathophysiological hallmark of NAFLD and it is believed that it is present in up to 95% of NAFLD patients. Insulin allows free fatty esterification and triglyceride fat storage in adipose tissue. When IR develops, free fatty acids (FFA) are inappropriately shifted to nonadipose tissue, such as the liver. IR increases FFA influx to the liver by decreased inhibition of lipolysis and also increased *de novo* intrahepatic synthesis of FFA-*de novo* lipogenesis. Furthermore, patients with NAFLD have decreased levels of “protective adipokine,” such as adiponectin as a consequence of IR. Normally, adiponectin inhibits liver gluconeogenesis and inhibits lipogenesis. Consequently, decreased adiponectin levels lead to fatty acid oxidation and increase fat accumulation in the liver. Enhanced oxidative stress and apoptosis can also contribute to the development and progression of NASH [14]. Furthermore, it has been shown that in comparison with patients without NAFLD, NAFLD patients have significantly higher levels of various plasma proinflammatory cytokines and procoagulations factors. Therefore, subchronic liver inflammation in NAFLD/NASH patients leads and contributes to dyslipidemia, inflammation, enhanced oxidative stress, and endothelial dysfunction.

Similarly, growing evidence suggests that the MS is an important factor in the pathogenesis of CKD. The best confirmation of this pathogenic link is hypertensive and diabetic nephropathy as the main causes of CKD. For example, a cross-sectional study of 574 nondiabetic patients showed that the prevalence of CKD was higher and mean estimated glomerular filtration rate (eGFR) was lower in patients who met the MS criteria compared with those who did not [19]. Furthermore, Ferraro et al. in their cross-sectional study which enrolled 3.757 individuals showed a direct correlation between the number of MS traits and nephropathy [20]. Similar results were reported by recent meta-analysis. In this work, an additional three studies reported an increased risk for development of microalbuminuria or overt proteinuria with MS [21]. MS is associated with inflammation in the general population and data from an epidemiological study based on the Third National Health and Nutrition Examination Survey (NHANES III) showed that such connection exists also in patients with CKD. The relevance of this is possible synergistic effects of MS and inflammation on the incidence of atherosclerotic events and thereby interventions toward MS might possibly modulate inflammation [22]. It is important to note that IR has been shown to be extensively linked to an increase in incidence of CKD. For example, data from the Cardiovascular Health Study that included 4680 adults showed that lower eGFR was associated with IR [23]. Furthermore, a study in total of 1456 Asian people conducted by Cheng at al. [24] observed that IR is associated with decline in renal function. Similar results were observed by some other authors. In patients with CKD the exact mechanism of IR is not known but it is believed that vitamin D deficiency, obesity, metabolic acidosis, inflammation, and accumulation of “uremic toxins” contribute to the development of IR [3]. Hyperlipidemia is one of the features of NAFLD and also contributes to the development of IR. Importantly, hyperlipidemia is also one of the challenging features to treat in individuals with CKD. As previously mentioned, the presence of IR is recognized as the pathophysiological hallmark of NAFLD. Furthermore, and similarly to the results observed in NAFLD patients, a number of studies have indicated that in CKD patients there are decreased adiponectin levels, increased oxidative stress, and elevated levels of proinflammatory cytokines and hypercoagulation [15, 17]. Concerning that many of these studies performed on humans are observational, further investigations that will verify whether treating the features of MS can prevent the development and progression of CKD are needed.

## 3. Chronic Kidney Disease and NAFLD

Growing evidence suggests that NAFLD may be linked to an increased risk for CKD. Several reports have investigated the association of CKD and NAFLD. Given the fact that about 90% of patients with NAFLD have more than one component of MS and 35–75% meet the diagnostic criteria, it is not surprising that NAFLD patients will have also decreased renal function. Recent studies that investigated the association between those two conditions are shown in Table 1.

Two cross-sectional studies conducted by Targher and colleagues [25, 26] showed that the prevalence of CKD was significantly higher among patients with ultrasound-defined NAFLD than in patients without steatosis. Another cross-sectional study conducted by Hwang et al. [27] analyzed 1361 patients who had an abnormal glucose tolerance test (prediabetes or newly diagnosed diabetes) on routine screening. They founded that patients with ultrasound-defined NAFLD had a significantly higher prevalence of microalbuminuria than those without this disease. A few large population-based studies showed that the presence of NAFLD is independently associated with increased prevalence of CKD. In those studies NAFLD was defined by elevated liver enzymes, alanine aminotransferase (ALT), or gamma-glutamyltransferase (GGT) [28, 29]. On the other hand, data from Framingham Heart Study did not find significant association between serum creatinine and GGT levels [30]. In all of these investigations NAFLD was detected by either liver enzymes or by liver ultrasound. Nevertheless, it is noteworthy to mention that aminotransferase levels which are used as a marker of liver damage are normal in approximately half of all patients with NAFLD. Therefore, normal values do not exclude NAFLD and liver fibrosis, so the results of these studies should be interpreted with caution. On the other hand, the sensitivity of the ultrasonography for detection of hepatic steatosis is between 93% and 100% if the fat content in hepatic parenchyma is >33%. It is a relatively subjective method and is therefore prone to sampling error.

A few, relatively small studies used liver biopsy for NAFLD diagnosis. For example, Yilmaz et al. [31] have found that patients with biopsy-proven NASH had moderately decreased eGFR values and higher frequency of abnormal albuminuria and CKD than matched control subjects. Also, they have found that severity of NASH histology was associated with decreased kidney function. Targher [33] and colleagues demonstrated significant association between severity of IR and microalbuminuria in 87 patients with biopsy-proven NAFLD. On the other hand, Manco et al. [32] did not find any association in markers of kidney function between overweight/obese children with biopsy-proven NAFLD and control, matched children without this disease. Although liver biopsy remains the gold standard in the diagnosis of NAFLD, it is an invasive procedure and is also prone to sampling error while still under discussion whether it is required to confirm a diagnosis of NAFLD. Also, liver biopsy cannot be performed routinely in everyday clinical practice and it cannot be performed in patients with impaired hemostasis, for example, in patients with advanced CKD [9–12]. Therefore, searching for new noninvasive tool for assessment of NAFLD is necessary. Recently, a novel parameter has been developed, that is, the controlled attenuation parameter (CAP), one that can be quantified using transient elastography (TE) (Fibroscan) and which is able to efficiently separate different grades of severity of steatosis. CAP is based on the properties of ultrasonic signals acquired by the Fibroscan. This diagnostic tool allows us to simultaneously measure liver stiffness and CAP in the same liver volume. The volume used for the measurement by the Fibroscan is 200 times larger than the one used for a liver biopsy specimen and therefore the usage in clinical practice becomes more frequent [36]. Recently we investigated the association between NAFLD and decreased kidney function. To this end we used TE (Fibroscan-CAP). We have found a high prevalence of NAFLD in patients with CKD stages III and IV. In our study the severity of liver steatosis was negatively correlated with kidney function [16]. On the other hand, the main problem of this noninvasive method, as well as with the other noninvasive tests that were investigated in patients with NAFLD/NASH, is the lack of a real gold standard to validate the tests. Therefore, continued research in this area will give us the opportunity to offer our patients more precise and noninvasive diagnostic tools. So far, liver biopsy will still probably be part of clinical practice in the coming years, but future biochemical and technique progress will challenge previously entrenched assumptions and will change our current approach to liver disease in the next decade. Consequently, all of these studies give clear evidence that NAFLD/NASH is associated with a greater prevalence of decreased kidney function and suggest that NAFLD is an important risk factor of CKD development and progression. However, the cross-sectional format of these studies does not allow conclusions whether the link between CKD and NAFLD is causal and should be interpreted with caution.

On the other hand, few prospective studies were assessing the link of NAFLD and the incidence of CKD [34, 35]. All of them found that NAFLD was associated with decreased kidney function. In these studies NAFLD was defined by ultrasound or by liver enzymes (GGT) and was not confirmed by liver biopsy. However, as it was previously mentioned, it would be unacceptable to perform liver biopsy routinely in large studies.

Despite all limitations in the abovementioned studies, data showed a significant association between NAFLD and the risk of incident CKD.

## 4. What Is the Possible Mechanism That Links NAFLD and Chronic Kidney Disease?

The underlying mechanism by which NAFLD increases the risk for development and progression of CKD remains to be elucidated. The most obvious explanation is that this association simply reflects the coexistence of known underlying cardiometabolic risk factors, features of MS. But we as well as other authors believe that NAFLD especially in its necro-inflammatory form (NASH) is, even partially, involved in CKD pathogenesis. The liver is the central organ for the production of various classical biomarkers of inflammation and endothelial dysfunction, the secretion of which partly depends on factors that are upregulated in the presence of IR and the MS. Today, there is growing evidence suggesting that in patients with NAFLD/NASH there are increased production and release of various proinflammatory cytokines. Therefore, systemic release of various promoters of inflammation, such as increased reactive oxygen species, TNF-*α*, TGF-*β*, plasminogen activator inhibitor-1, C-reactive protein (CRP), and IL-6, produced by hepatocytes and nonparenchymal cells, including Kupffer cells and hepatic stellate cells, can be possible mediators that link NAFLD and CKD. In the context of CKD, preliminary evidence in animal models suggests that cytokine imbalance may contribute to the pathogenesis of CKD [17, 37]. Furthermore, NAFLD/NASH can exacerbate hepatic/systemic IR and be a factor for further increase in whole-body IR and promote the development of atherogenic dyslipidemia. Consequently, this will play an important role in CKD development and progression.

Other pathophysiological mechanisms that are not strictly related to liver inflammation could also link NAFLD and CKD. Namely, decreased levels of plasma adiponectin may represent another potential mechanism that links these two conditions. This observation is supported by a recent review of animal and human models by Ix and Sharma [38]. In their hypothesis, the link occurs between liver, kidney, and adipose tissue through an interorgan communication orchestrated by fetuin-A and adiponectin. In liver and kidney, lower adiponectin levels reduce activation of the energy sensor 5′-AMP activated protein kinase (AMPK), which is pivotal to direct podocytes and hepatocytes to compensatory and potentially deleterious pathways, leading to inflammatory and profibrotic cascades culminating in end-organ damage (liver cirrhosis and ESRD).

Finally, another possible factor linking NAFLD, microalbuminuria, and IR is the rennin-angiotensin system (RAS). It is believed that RAS could be involved in the development of IR and it seems that it promotes hepatic fibrogenesis [31, 39, 40].

According to all of these observations, further studies that will investigate the possible pathophysiological link between liver and kidney that will reveal specific mechanism by which NAFLD might contribute to the pathogenesis of CKD are needed.

## 5. Conclusion

Accumulative body of evidence has shown an increase in the incidence of CKD in subjects with NAFLD. It is reasonable to suggest that an increase in the epidemic of NAFLD may represent a potential for an increase in the incidence of CKD. Common factors underlying the pathogenesis of NAFLD and CKD may be insulin resistance, oxidative stress, activation of rennin-angiotensin system, and inappropriate secretion of inflammatory cytokines by steatotic and inflamed liver. Further studies that will investigate the possible link between liver and kidney are needed. It is important to note that NAFLD patients require a multidisciplinary approach for their treatment. Therefore, physicians who manage those patients should not only focus on liver disease but should also recognize extra-hepatic manifestations of NAFLD. On the other hand, physicians who manage patients with impaired renal function should also recognize those patients having NAFLD. This could consequently help to recognize high risk patients who should be aggressively treated in order to reduce their morbidity and mortality.

## Figures and Tables

**Table 1:** Cross-sectional and prospective studies of the association between chronic kidney disease and NAFLD.

Authors	Study population	Diagnosis of NAFLD	Study measures	Main findings
Targher et al. [25]	2103 diabetic patients	Ultrasound	eGFR ≤ 60 mL/min/1.73 m^2^ or over proteinuria	The prevalence of CKD was higher among patients with NAFLD than among without NAFLD (P < 0.0001)

Targher et al. [26]	202 type 1 diabetic patients	Ultrasound	eGFR ≤ 60 mL/min/1.73 m^2^ or urinary alb/creat ratio ≥ 30 mg/g	NAFLD is associated with increased risk of prevalent CKD

Hwang et al. [27]	1.361 patients with impaired glucose tolerance or newly diagnosed diabetes	Ultrasound	Urinary alb/creat ratio ≥ 30 mg/g	NAFLD is associated with increased risk of prevalent microalbuminuria

Targher et al. [28]	National Health and Nutritional Examination Survey (NHANES) 2001–2006; involved 13188 patients	Liver enzymes (GGT)	eGFR ≤ 60 mL/min/1.73 m^2^ or urinary alb/creat ratio ≥ 30 mg/g	Elevated serum levels of GGT were associated with prevalent CKD

Yun et al. [29]	Health-examination survey of 37085 individuals	Liver enzymes (ALT)	eGFR	Individuals with ALT > 40 U/L had lower eGFR than those with ALT < 40 U/L

Lee et al. [30]	Framingham Heart Study; involved 3451 subjects	Liver enzymes (GGT)	Serum creatinine	No differences

Mikolasevic et al. [16]	75 patients with CKD stage III and IV	Transient elastography (Fibroscan-CAP)	eGFR ≤ 60 mL/min/1.73 m^2^	High prevalence of NAFLD in CKD patients. The severity of liver steatosis (defined by CAP values) was negatively correlated with kidney function

Yilmaz et al. [31]	87 NAFLD patients	Biopsy	24 hrs urinary albumin excretion rate	Microalbuminuria is associated with IR and liver fibrosis in NAFLD patients

Manco et al. [32]	80 overweight or obese children with NAFLD and 59 age- and sex-matched children	Biopsy	Creatinine clearance and 24 hr urinary albumin excretion rate	No difference

Targher et al. [33]	80 NASH patients matched with age-, sex- and BMI matched subjects without steatosis	Biopsy	eGFR ≤ 60 mL/min/1.73 m^2^ or urinary alb/creat ratio ≥ 30 mg/g	NASH patients had higher frequency of CKD

Targher et al. [34]	Valpolicella Heart Diabetes Study; 1760 type 2 diabetic patients with normal kidney function; length of follow-up: 6.5 years	Ultrasonography	eGFR ≤ 60 mL/min/1.73 m^2^ or overt proteinuria	NAFLD is associated with increased risk of incident CKD

Ryu et al. [35]	10.337 non-diabetic and non-hypertensive patients with normal kidney function; length of followup: 2.5 years	Liver enzymes (GGT)	eGFR ≤ 60 mL/min/1.73 m^2^ or overt proteinuria	Elevated serum GGT levels are associated with increased risk of incident CKD

Estimated glomerular filtration rate (eGFR); nonalcoholic fatty liver disease (NAFLD); albumin (Alb); creatinine (Creat); alanine aminotransferase (ALT); gamma-glutamyltransferase (GGT); body mass index (BMI); controlled attenuation parameter (CAP).
